# Supplementary material for: Feasibility of high-resolution perfusion imaging using arterial spin labeling MRI at 3 Tesla
Source: Front Physiol. 2024 Jan 3;14:1271254. doi: 10.3389/fphys.2023.1271254 (PMC10791866; doi:10.3389/fphys.2023.1271254)
Supplement: Supplementary file 1 [file DataSheet1.PDF]

frontiers

Supplementary Material

Impact of deblur options on the 3D-GRASE ASL data

	FFT			Lucy	
	**Direct**	**Lorentz**	**Lorwien**	**Direct**	**Lorentz**
Clinical	6.38 ± 0.33	6.57 ± 0.3	6.65 ± 0.33	6.55 ± 0.3	6.55 ± 0.35
Hires	2.38 ± 0.13	2.53 ± 0.12	2.43 ± 0.14	2.54 ± 0.09	2.62 ± 0.09

Table S1. Table of FWHM (in mm) estimated using AFNI's *3dFWHMx* for different deblurring methods available in $oxasl\_deblur$. Numerical values presented are mean ± std. dev across participants. This evaluation was carried out on data from the 32 ch coil only.

Comparison of clinical and hires pCASL protocols

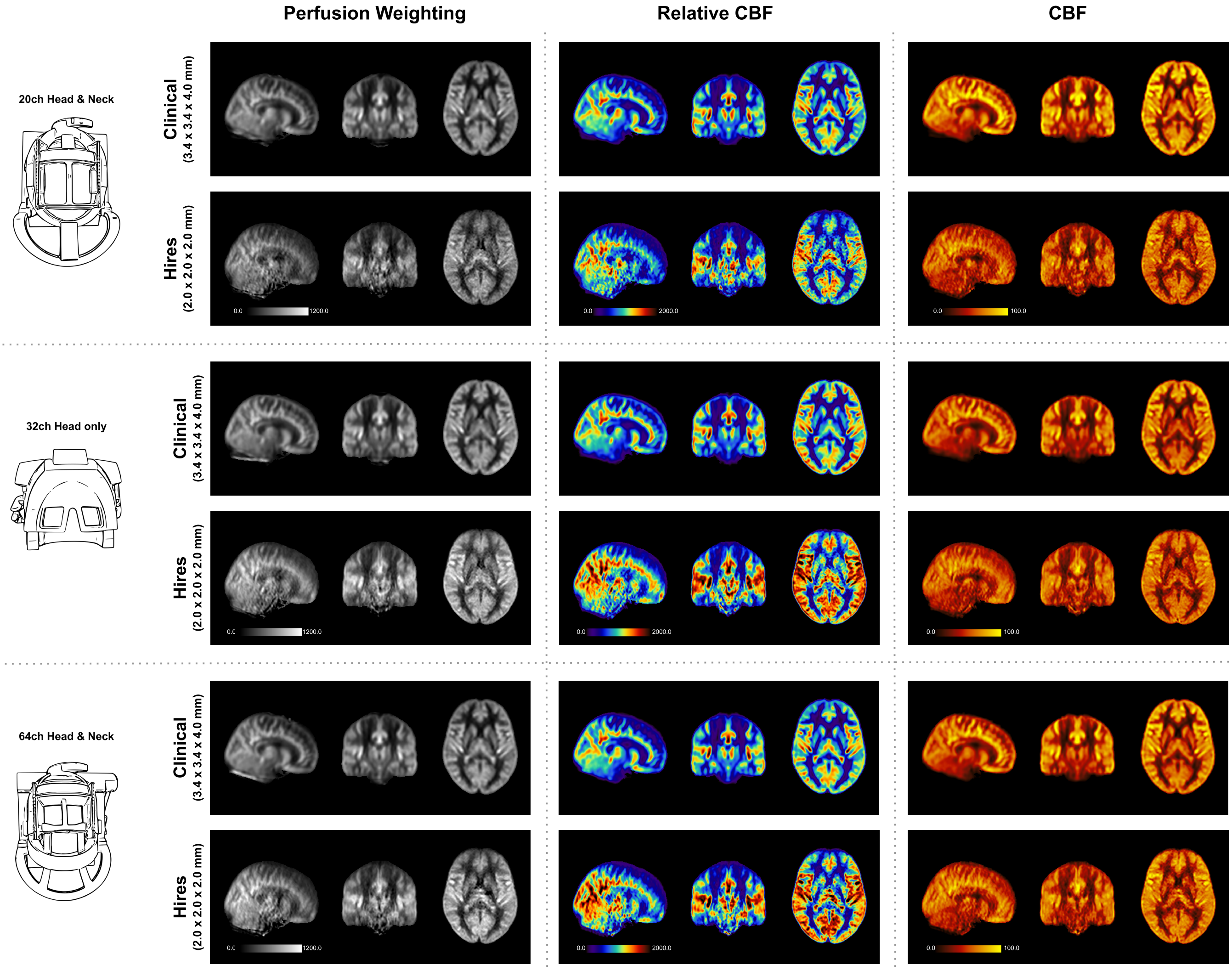

Figure S1: Mosaic of orthogonal views of the group average perfusion-weighted (MRI signal units), relative CBF (arbitrary units) and CBF (mL/100g/min) maps for data acquired using the three respective head coils.. In each panel, the maps obtained from the clinical and hires pCASL acquisitions are displayed in the top and bottom rows, respectively.

Tables of Summary Statistics : Clinical vs Hires pCASL

CBF (mL/100 g/min)
pCASL Clinical

Statistic	20 ch	32 ch	64 ch
Mean	72.57	68.43	67.36
Median	70.81	65.42	64.42
Standard Deviation	27.85	27.96	27.61
Inter-quartile Range	36.85	37.70	37.12
1st Quartile	53.55	48.65	47.79
3rd Quartile	90.40	86.35	84.91
Outlier Percentage	0.79%	0.80%	0.88%

CBF (mL/100 g/min)
pCASL Hires

Statistic	20 ch	32 ch	64 ch
Mean	57.90	58.30	59.56
Median	56.55	56.66	57.75
Standard Deviation	26.15	25.07	25.77
Inter-quartile Range	35.58	33.59	34.85
1st Quartile	39.42	40.83	41.44
3rd Quartile	75.00	74.42	76.28
Outlier Percentage	0.69%	0.78%	0.74%

Table S2. Summary Statistics of quantitative CBF (in mL/100 g/min) for Clinical and Hires pCASL data over all subjects in the study

Relative CBF
pCASL Clinical

Statistic	20 ch	32 ch	64 ch
Mean	960.79	1016.20	1001.84
Median	933.14	963.99	950.55
Standard Deviation	385.91	435.08	433.61
Inter-quartile Range	533.11	566.97	574.38
1st Quartile	678.91	704.31	687.94
3rd Quartile	1212.03	1271.28	1262.31
Outlier Percentage	0.81%	1.66%	1.46%

Relative CBF
pCASL Hires

Statistic	20 ch	32 ch	64 ch
Mean	944.67	1072.07	1097.49
Median	885.92	1003.30	1023.64
Standard Deviation	502.92	561.64	578.83
Inter-quartile Range	693.79	759.00	788.37
1st Quartile	568.31	656.43	666.43
3rd Quartile	1262.09	1415.43	1454.80
Outlier Percentage	0.99%	1.28%	1.18%

Table S3. Summary Statistics of relative CBF for Clinical and Hires pCASL data over all subjects in the study

Perfusion weighting (a. u.)
pCASL Clinical

Statistic	20 ch	32 ch	64 ch
Mean	575.50	608.55	600.29
Median	557.94	576.44	568.68
Standard Deviation	226.10	256.24	256.66
Inter-quartile Range	312.50	334.30	338.79
1st Quartile	409.56	424.01	414.27
3rd Quartile	722.06	758.30	753.05
Outlier Percentage	0.84%	1.69%	1.51%

Perfusion weighting (a. u.)
pCASL Hires

Statistic	20 ch	32 ch	64 ch
Mean	534.89	605.91	620.15
Median	500.93	565.98	577.39
Standard Deviation	280.25	313.30	322.90
Inter-quartile Range	386.34	423.67	440.03
1st Quartile	324.63	373.16	378.73
3rd Quartile	710.97	796.82	818.77
Outlier Percentage	1.03%	1.31%	1.21%

Table S4. Summary Statistics of Perfusion-weighting (in arbitrary units) for Clinical and Hires pCASL data over all subjects in the study

Perfusion tSNR
pCASL Clinical

Statistic	20 ch	32 ch	64 ch
Mean	5.66	5.77	5.92
Median	5.43	5.51	5.64
Standard Deviation	2.35	2.45	2.53
Inter-quartile Range	3.06	3.17	3.23
1st Quartile	3.99	4.03	4.14
3rd Quartile	7.06	7.20	7.37
Outlier Percentage	1.50%	1.67%	1.83%

Perfusion tSNR
pCASL Hires

Statistic	20 ch	32 ch	64 ch
Mean	1.59	2.14	2.27
Median	1.45	1.85	1.97
Standard Deviation	0.92	1.39	1.44
Inter-quartile Range	1.16	1.63	1.74
1st Quartile	0.93	1.16	1.24
3rd Quartile	2.09	2.79	2.98
Outlier Percentage	2.29%	3.55%	3.20%

Table S5. Summary Statistics of perfusion tSNR for Clinical and Hires pCASL data over all subjects in the study

Perfusion tSNR
pCASL Clinical

Statistic	20 ch	32 ch	64 ch
Mean	5.66	5.77	5.92
Median	5.43	5.51	5.64
Standard Deviation	2.35	2.45	2.53
Inter-quartile Range	3.06	3.17	3.23
1st Quartile	3.99	4.03	4.14
3rd Quartile	7.06	7.20	7.37
Outlier Percentage	1.50%	1.67%	1.83%

Perfusion tSNR (volume-scaled)
pCASL Hires

Statistic	20 ch	32 ch	64 ch
Mean	9.19	12.35	13.11
Median	8.40	10.66	11.40
Standard Deviation	5.30	8.03	8.34
Inter-quartile Range	6.70	9.42	10.03
1st Quartile	5.38	6.70	7.18
3rd Quartile	12.08	16.12	17.21
Outlier Percentage	2.29%	3.55%	3.20%

Table S6. Summary Statistics of perfusion tSNR after scaling the pCASL hires data in Table S7 by the ratio of voxel-volumes.

Comparison of Hires pCASL and PASL protocols

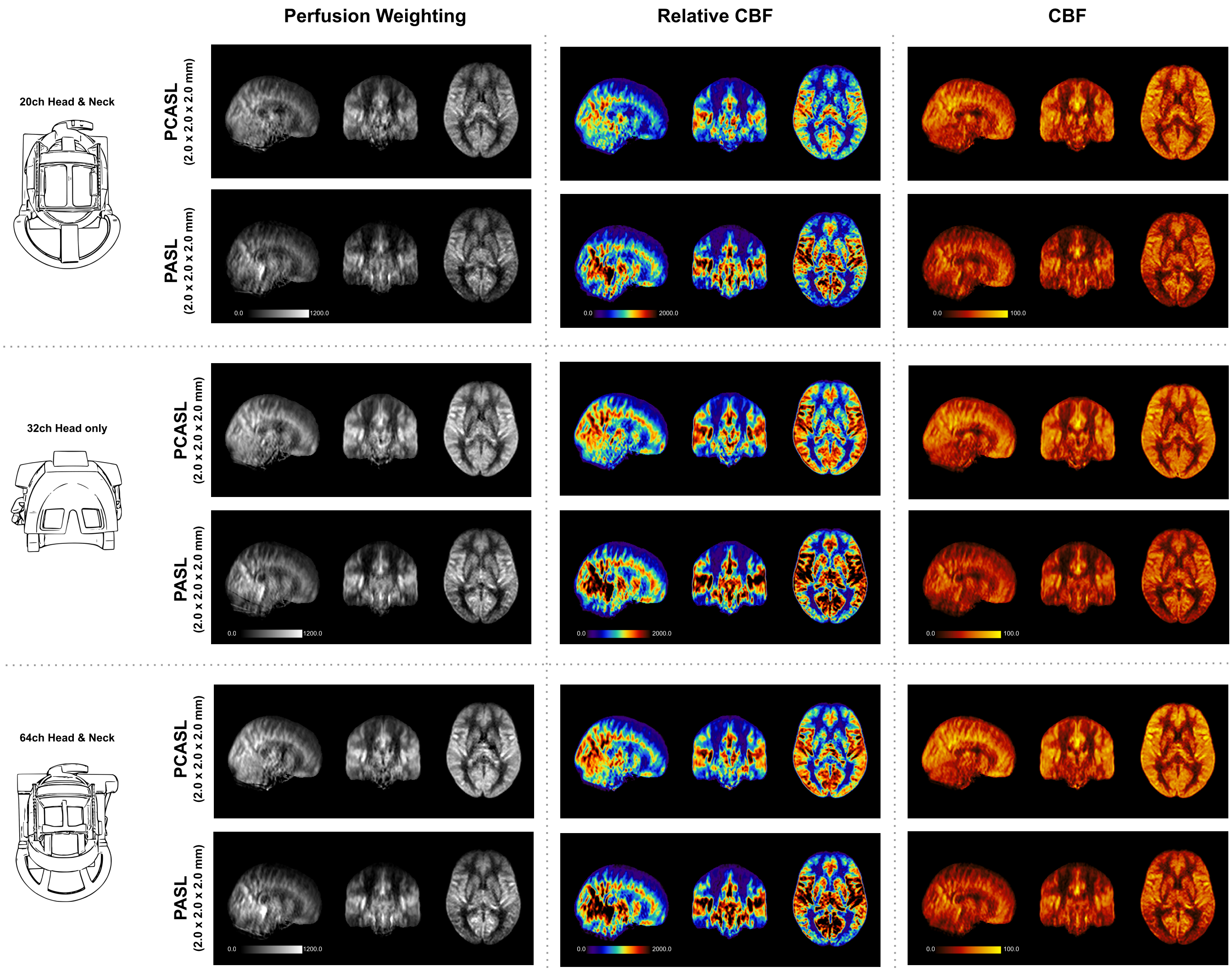

Figure S2: Mosaic of orthogonal views of the group average perfusion-weighted (MRI signal units), relative CBF (arbitrary units) and CBF (mL/100g/min) maps for data acquired using the three respective head coils. In each panel, the maps obtained from the hires pCASL and PASL acquisitions are displayed in the top and bottom rows, respectively.

Impact of head coil choice on hires acquisitions

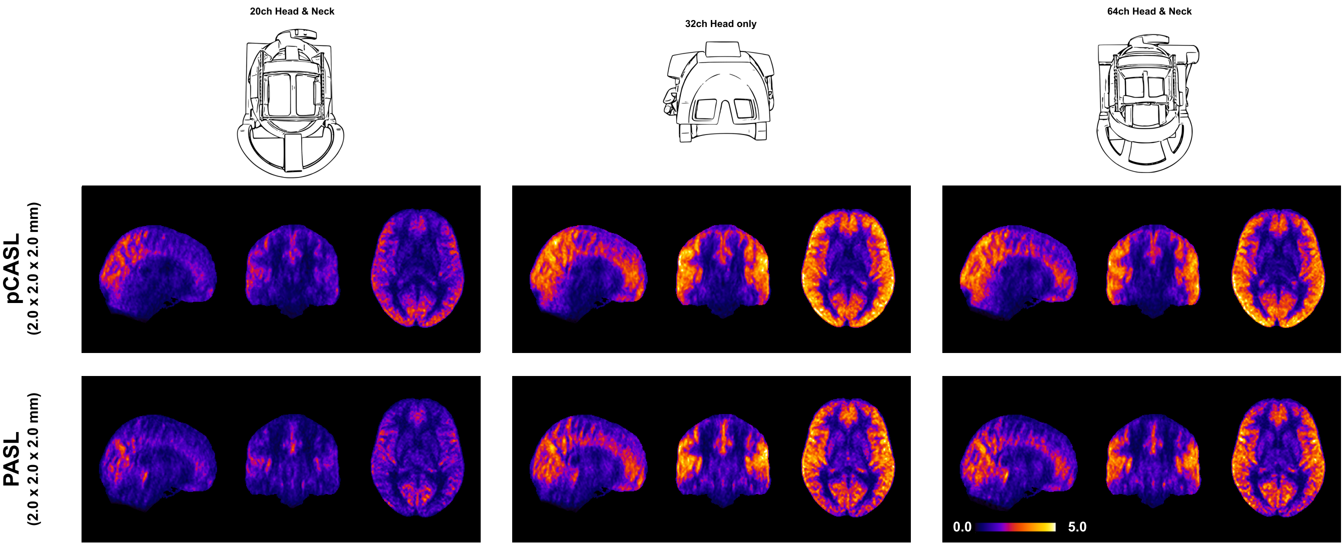

Figure S3: Orthogonal views of the group average perfusion tSNR maps for the hires acquisitions with pCASL and PASL labelling schemes.

Tables of Summary Statistics : Hires pCASL vs Hires PASL

CBF (mL/100 g/min)
pCASL Hires

Statistic	20 ch	32 ch	64 ch
Mean	57.90	58.30	59.56
Median	56.55	56.66	57.75
Standard Deviation	26.15	25.07	25.77
Inter-quartile Range	35.58	33.59	34.85
1st Quartile	39.42	40.83	41.44
3rd Quartile	75.00	74.42	76.28
Outlier Percentage	0.69%	0.78%	0.74%

CBF (mL/100 g/min)
PASL Hires

Statistic	20 ch	32 ch	64 ch
Mean	42.06	42.12	42.37
Median	39.65	39.82	40.20
Standard Deviation	25.94	25.71	25.80
Inter-quartile Range	37.10	36.93	37.09
1st Quartile	21.86	22.11	22.30
3rd Quartile	58.96	59.04	59.39
Outlier Percentage	0.77%	0.72%	0.71%

Table S7. Summary Statistics of quantitative CBF (in mL/100 g/min) for pCASL Hires and PASL Hires data over all subjects in the study

Relative CBF
pCASL Hires

Statistic	20 ch	32 ch	64 ch
Mean	944.67	1072.07	1097.49
Median	885.92	1003.30	1023.64
Standard Deviation	502.92	561.64	578.83
Inter-quartile Range	693.79	759.00	788.37
1st Quartile	568.31	656.43	666.43
3rd Quartile	1262.09	1415.43	1454.80
Outlier Percentage	0.99%	1.28%	1.18%

Relative CBF
PASL Hires

Statistic	20 ch	32 ch	64 ch
Mean	988.63	1114.79	1126.50
Median	863.30	970.60	984.09
Standard Deviation	701.69	793.27	801.86
Inter-quartile Range	978.52	1103.48	1121.65
1st Quartile	435.20	489.73	492.45
3rd Quartile	1413.71	1593.21	1614.10
Outlier Percentage	1.32%	1.38%	1.30%

Table S8. Summary Statistics of relative CBF for pCASL Hires and PASL Hires data over all subjects in the study

Perfusion weighting (a. u.)
pCASL Hires

Statistic	20 ch	32 ch	64 ch
Mean	534.89	605.91	620.15
Median	500.93	565.98	577.39
Standard Deviation	280.25	313.30	322.90
Inter-quartile Range	386.34	423.67	440.03
1st Quartile	324.63	373.16	378.73
3rd Quartile	710.97	796.82	818.77
Outlier Percentage	1.03%	1.31%	1.21%

Perfusion weighting (a. u.)
PASL Hires

Statistic	20 ch	32 ch	64 ch
Mean	420.22	471.74	476.20
Median	369.42	412.66	417.72
Standard Deviation	286.37	324.00	327.51
Inter-quartile Range	396.12	447.66	454.97
1st Quartile	196.43	218.29	219.09
3rd Quartile	592.56	665.95	674.06
Outlier Percentage	1.38%	1.44%	1.36%

Table S9. Summary Statistics of Perfusion-weighting (in arbitrary units) for pCASL Hires and PASL Hires data over all subjects in the study

Perfusion tSNR
pCASL Hires

Statistic	20 ch	32 ch	64 ch
Mean	1.59	2.14	2.27
Median	1.45	1.85	1.97
Standard Deviation	0.92	1.39	1.44
Inter-quartile Range	1.16	1.63	1.74
1st Quartile	0.93	1.16	1.24
3rd Quartile	2.09	2.79	2.98
Outlier Percentage	2.29%	3.55%	3.20%

Perfusion tSNR
PASL Hires

Statistic	20 ch	32 ch	64 ch
Mean	1.26	1.74	1.84
Median	1.09	1.41	1.51
Standard Deviation	0.90	1.38	1.42
Inter-quartile Range	1.15	1.62	1.73
1st Quartile	0.58	0.74	0.79
3rd Quartile	1.73	2.36	2.52
Outlier Percentage	2.47%	3.81%	3.30%

Table S10. Summary Statistics of perfusion tSNR for pCASL Hires and PASL Hires data over all subjects in the study

Perfusion tSNR (volume-scaled)
pCASL Hires

Statistic	20 ch	32 ch	64 ch
Mean	9.19	12.35	13.11
Median	8.40	10.66	11.40
Standard Deviation	5.30	8.03	8.34
Inter-quartile Range	6.70	9.42	10.03
1st Quartile	5.38	6.70	7.18
3rd Quartile	12.08	16.12	17.21
Outlier Percentage	2.29%	3.55%	3.20%

Perfusion tSNR (volume-scaled)
PASL Hires

Statistic	20 ch	32 ch	64 ch
Mean	7.28	10.06	10.63
Median	6.28	8.12	8.72
Standard Deviation	5.18	8.00	8.24
Inter-quartile Range	6.63	9.39	10.02
1st Quartile	3.37	4.25	4.54
3rd Quartile	10.00	13.64	14.56
Outlier Percentage	2.47%	3.81%	3.30%

Table S11. Summary Statistics of perfusion tSNR after scaling the pCASL hires data in Table S7 by the ratio of voxel-volumes.

Single participant maps for all three ASL protocols

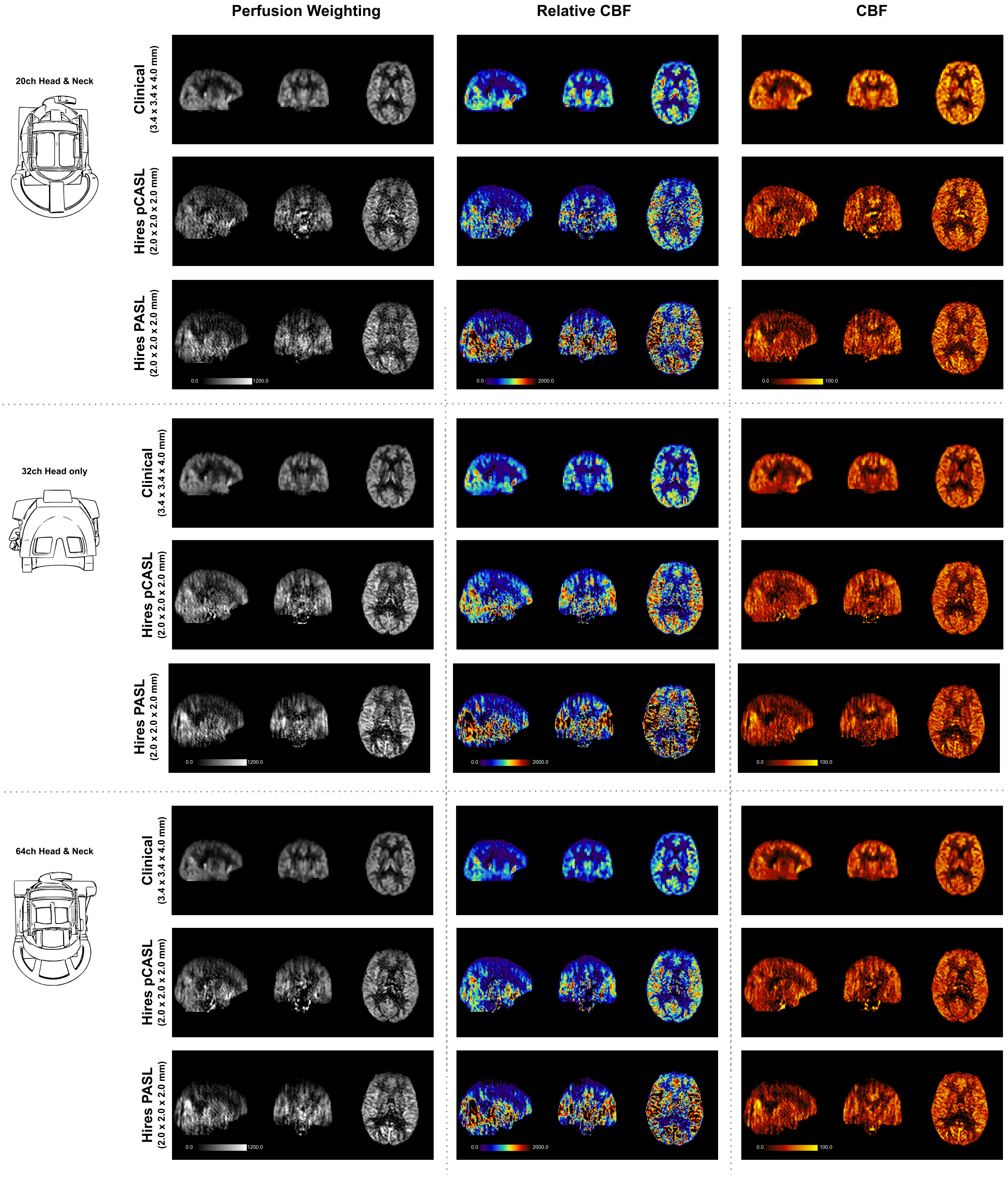

Figure S4: Mosaic of orthogonal views of a single participant's perfusion-weighted (MRI signal units), relative CBF (arbitrary units) and CBF (mL/100g/min) maps for data acquired using the three respective head coils. In each panel, the maps obtained from the clinical pCASL, hires pCASL and hires PASL acquisitions are displayed in the top, middle and bottom rows, respectively.
